# Children's Food and Management

**Published:** 1886-11-20

**Authors:** 


### Till the Doctor Comes.

[Inquiries and Communications for this column should be addressed, "Physician," care of the Editor of The Hospital.]

### IV.—CHILDREN'S FOOD AND MANAGEMENT.

It is terrible to think of the enormous sacrifice of infant life that takes place every year because parents, or those in charge of children, neglect to feed and tend them properly. In ninety cases out of a hundred the child is born healthy, and, if properly reared, would develop into a robust and vigorous adult. In the middle classes, and especially the upper middle classes, too much fuss is often made, and children become delicate because they are crippled by over-tending. Abundance of light, air, and nourishing food are essential. Yet in London, for instance, how much air and light are the children allowed? The nurseries (day and night) are usually at the top of the house, and are—more often than not—confined, low, and ill-ventilated apartments, in which it is humanly impossible for a child to progress satisfactorily. As to food, it is sad to reflect how many mothers are wholly ignorant of the art of feeding a little one. When the child becomes less and less vigorous, it is regarded as a sickly member of the family, and medicine is prescribed, instead of the change of diet and healthful regimen needed. If a child cries, it is either frightened, in pain, or hungry, and this fact should not be lost sight of by mothers. It is scarcely too much to say that if children are brought up under a good system, if they are fed upon good, plain, wholesome food, and if their sleeping accommodation is adequate, they will thrive almost as well in London as anywhere else.

**Good Food!** By good food is meant suitable food properly cooked. For a baby not fed by the mother, condensed milk, rightly mixed, is most nourishing. When condensed milk does not appear to agree with the child, then inquiry will almost certainly show that the fault lies in the mixing, and not in the condensed milk. The constitution of one child differs from that of another, and this fact must keep the careful nurse on her guard, to anticipate and provide for any peculiarities of diet which may be necessary in consequence. Remembering this, it may be broadly asserted that a healthy child will do well on condensed milk alone for the first three months. Afterwards must be added by degrees biscuit powder, or some other similar food, to be followed in from three to six months by beef tea. When meat is given at first, raw rumpsteak, finely minced and cooked by means of boiling gravy poured over it, should be given in small quantities. When meat in larger quantities is required, none but that which has been once cooked only should be given to children. They cannot digest twice-cooked food, and it ought never to be given to them.

**Milk and Village Children.** In this connection attention may usefully be called to the cruelty practised upon the children of the poor by certain landowners and farmers, who refuse to supply the poor with milk. Notice was recently called in *The Lancet* to the action of a wealthy landlord, who behaved with brutal tyranny to the poor on his estate. Here, in three weeks, out of a population of 100 souls, three children are reported to have died from want of that nourishment which good milk can alone supply. In one of these cases the parents resided under the same roof that covered the squire's dairy, wherein was stored the milk of a large herd of cows. Not one drop of milk was, however, allowed to be given to the poor suffering little one ; nor in this village can the poor procure milk. Now and again buttermilk was occasionally given in small quantities, but since the *Lancet* exposed the landlord's cruelty this practice is forbidden. In this village the diet of the babies had, in consequence, to consist of corn flour, bread and water, to their great danger and enfeeblement. It would be a great gain if the clergy in each parish were to arrange to supply milk from the school or some other centre. The present state of things is often a disgrace to our common humanity, and a remedy should speedily be found for it.

*(To be continued.)*

